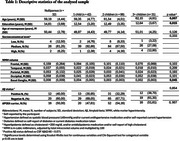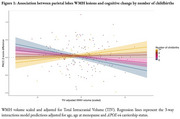# Exploring the impact of childbirth on the association between white matter hyperintensity load and cognitive changes in post‐menopausal women at risk of Alzheimer's Disease

**DOI:** 10.1002/alz70856_100808

**Published:** 2025-12-25

**Authors:** Clara Gallay, Jordi Huguet, Armand González Escalante, Henrik Zetterberg, Kaj Blennow, Ana Fernández‐Arcos, Marc Suárez‐Calvet, Erin E. Sundermann, Juan Domingo Gispert, Oriol Grau‐Rivera, Anna Brugulat‐Serrat

**Affiliations:** ^1^ Barcelonaβeta Brain Research Center (BBRC), Pasqual Maragall Foundation, Barcelona, Spain; ^2^ University Pompeu Fabra, Barcelona, Barcelona, Spain; ^3^ BarcelonaBeta Brain Research Center (BBRC), Barcelona, Spain; ^4^ Universitat Pompeu Fabra, Barcelona, Spain; ^5^ Hospital del Mar Research Institute (IMIM), Barcelona, Spain; ^6^ Department of Psychiatry and Neurochemistry, Institute of Neuroscience and Physiology, the Sahlgrenska Academy, University of Gothenburg, Molndal, Sweden; ^7^ University College London, London, United Kingdom, London, United Kingdom; ^8^ Institute of Neuroscience and Physiology, University of Gothenburg, Gothenburg, Mölndal, Sweden; ^9^ UCL Institute of Neurology, London, United Kingdom; ^10^ Institute of Neuroscience and Physiology, Sahlgrenska Academy, University of Gothenburg, Gothenburg, Sweden; ^11^ Institute of Neuroscience and Physiology, Department of Psychiatry and Neurochemistry, The Sahlgrenska Academy at University of Gothenburg, Mölndal, Sweden; ^12^ IMIM (Hospital del Mar Medical Research Institute), Barcelona, Spain; ^13^ Servei de Neurologia, Hospital del Mar, Barcelona, Spain; ^14^ Barcelonaβeta Brain Research Center (BBRC), Barcelona, Spain; ^15^ University of California, San Diego, La Jolla, CA, USA; ^16^ CIBER Bioingeniería, Biomateriales y Nanomedicina (CIBER‐BBN), Madrid, Spain; ^17^ Barcelona?eta Brain Research Center (BBRC), Pasqual Maragall Foundation, Barcelona, Spain; ^18^ Centro de Investigación Biomédica en Red de Fragilidad y Envejecimiento Saludable (CIBERFES), Instituto de Salud Carlos III, Barcelona, Spain; ^19^ Global Brain Health Institute, San Francisco, CA, USA; ^20^ Hospital del Mar Research Institute, Barcelona, Spain

## Abstract

**Background:**

Cerebrovascular diseases impact cognition in middle‐aged individuals. Hormonal changes caused by childbirth impact the cerebrovascular system, yet little is known about its influence on cognition. We examined the influence of associations between number of childbirths and white matter hyperintensities (WMH) on cognitive change, in cognitively unimpaired (CU) postmenopausal women at increased risk of AD.

**Method:**

Our sample includes 201 CU postmenopausal women from the ALFA+ study, enriched in AD risk factors. Regional values of WMH volumes were obtained averaging lesions within five regions: frontal, temporal, parietal and occipital lobes, and basal ganglia. Amyloid (Aβ) positivity was defined as CSF Aβ42/40 ratio<0.071. Cognitive performance was measured using PACC change within ±3 years. We conducted linear regression models to assess the effect of WMH (model 1) and childbirth (model 2) on PACC longitudinal change, including Aβ status as a covariate. A three‐way interaction between WMH, childbirth and Aβ status was then introduced to understand the role of AD pathology (model 3). All models included *APOE*‐ε4 carriership status, age, age at menopause, socio‐economical level and education as covariates (Table 1).

**Result:**

We did not find a significant main effect of WMH (model 1) or childbirth (model 2) as predictors of PACC change. A trending effect of Aβ status was observed in each case, where Aβ positivity was associated with steeper cognitive decline. In model 3, the number of childbirths significantly modified the association between PACC score and WMH volume in the parietal lobes (*p* = 0.019), suggesting more childbirths were linked with lesser cognitive change in women with higher WMH load. The three‐way interaction itself was not significant.

**Conclusion:**

In CU post‐menopausal women with no history of cardiovascular diseases, WMH volume and number of childbirths were not independently associated with cognitive outcome at 3 years. However, higher number of childbirths moderated the association between WMH and cognitive changes: women with higher WMH volume in the parietal lobes showed lesser cognitive decline, independently of amyloid burden. Results indicate that the impact of childbirth on cerebrovascular disease may be traceable decades after childbirth. Further studies are needed to investigate potential mechanisms linking pregnancy history with WMH and cognitive performance.